# Microbes

**Published:** 1889-10-12

**Authors:** 


### MICROBES.

In the last Croonian lectures, by Dr. Lauder Brunton, on "The Relationship between Chemical Structure and Physiological Action," the lecturer refers to the action of microbes on the intestines. The lecturer asserts that much of the diarrhœa of children is due to the formation of "tyrotoxicon" from decomposition of milk in the intestines. But one of the most fruitful sources of diarrhœa in children is the use of feeding-bottles with long tubes, which are generally imperfectly cleaned, so that when the milk is put perfectly fresh into the bottle it becomes inoculated with bacteria before it reaches the child's stomach. Dr. Brunton thinks there is little doubt that epidemic diarrhœa, choleraic diarrhœa, and Asiatic cholera are all due to microbes, although the nature of the microbe is not definitely settled in each case.

